# Single-cell analyses unravel cell type–specific responses to a vitamin D analog in prostatic precancerous lesions

**DOI:** 10.1126/sciadv.abg5982

**Published:** 2021-07-30

**Authors:** Mohamed A. Abu el Maaty, Elise Grelet, Céline Keime, Anna-Isavella Rerra, Justine Gantzer, Camille Emprou, Julie Terzic, Régis Lutzing, Jean-Marc Bornert, Gilles Laverny, Daniel Metzger

**Affiliations:** 1 Institut de Génétique et de Biologie Moléculaire et Cellulaire, Illkirch, France.; 2 Centre National de la Recherche Scientifique, UMR7104, Illkirch, France.; 3 Institut National de la Santé et de la Recherche Médicale (INSERM), U1258, Illkirch, France.; 4 Université de Strasbourg, Illkirch, France.

## Abstract

Epidemiological data have linked vitamin D deficiency to the onset and severity of various cancers, including prostate cancer, and although in vitro studies have demonstrated anticancer activities for vitamin D, clinical trials provided conflicting results. To determine the impact of vitamin D signaling on prostatic precancerous lesions, we treated genetically engineered Pten^(i)pe−/−^ mice harboring prostatic intraepithelial neoplasia (PIN) with Gemini-72, a vitamin D analog with reported anticancer activities. We show that this analog induces apoptosis in senescent PINs, normalizes extracellular matrix remodeling by stromal fibroblasts, and reduces the prostatic infiltration of immunosuppressive myeloid-derived suppressor cells. Moreover, single-cell RNA-sequencing analysis demonstrates that while a subset of luminal cells expressing Krt8, Krt4, and Tacstd2 (termed luminal-C cells) is lost by such a treatment, antiapoptotic pathways are induced in persistent luminal-C cells. Therefore, our findings delineate the distinct responses of PINs and the microenvironment to Gemini-72, and shed light on mechanisms that limit treatment’s efficacy.

## Teaser

Anti- and protumoral effects of a vitamin D analog revealed at single-cell resolution.

## INTRODUCTION

Prostate cancer (PCa) is the second most common cancer affecting men worldwide and the first in the United States (*1*, *2*). The initial step in the malignant transformation of prostatic epithelial cells is the development of precancerous lesions termed prostatic intraepithelial neoplasia (PIN), which may progress to adenocarcinoma and become metastatic (*3*). Localized PCa can be cured by radical prostatectomy or radiotherapy (*3*), but these interventions may induce side effects. Metastatic PCa is treated by androgen deprivation therapy, but although initially effective, progression to castration-resistant PCa occurs in most patients (*3*), for whom few treatment options are available. Therefore, effective preventive or therapeutic strategies are highly desired to combat PCa.

Vitamin D3 is a seco-steroid with pleiotropic effects in mammals (*4*). Its active form, 1,25-dihydroxyvitamin D3 [1,25(OH)_2_D_3_], plays a key role in calcium homeostasis (*4*). Moreover, it exerts immunomodulating and antitumoral effects in various preclinical models (*4*–*6*), and numerous epidemiological reports have linked vitamin D deficiency to various forms of cancer (*5*). Experimental studies have reported various mechanisms by which 1,25(OH)_2_D_3_ may target tumor cells, including inhibition of proliferation and induction of apoptosis (*5*).

Although the large-scale VITAL study concluded that a daily high dose of vitamin D3 (2000 IU) does not reduce the incidence of colorectal, breast, or prostate cancer (*7*), subgroup analysis indicated that vitamin D supplementation may reduce the incidence of cancer in individuals with a body mass index lower than 25 kg/m^2^ (*7*). Moreover, studies conducted on human PCa cell lines have reported overwhelmingly positive results for 1,25(OH)_2_D_3_. It reduces the expression of antiapoptotic Bcl2 family members leading to apoptosis in LNCaP cells (*5*). In line with these results, a 4-month 1,25(OH)_2_D_3_ treatment has been shown to inhibit PIN formation in genetically engineered mice (*8*). However, this treatment regimen had no effect on high-grade PIN evolution (*8*), and a 3.5-month treatment increased the incidence of distant organ metastasis in a more aggressive PCa mouse model (*9*). On the other hand, a dose-dependent increase in dietary vitamin D led to a reduction in prostate adenocarcinoma incidence in mice (*10*). Thus, the impact of 1,25(OH)_2_D_3_ on PCa appears to be complex and stage dependent. Recent findings of single-cell RNA sequencing (scRNA-seq) studies of human and mouse prostates demonstrated the heterogeneity in the epithelial and stromal compartments (*11*–*13*). Because 1,25(OH)_2_D_3_ is known to target multiple cell types (*4*), elucidating its impact on the complex microenvironment of prostatic precancerous lesions may shed light on antitumor mechanisms and limitations of these regimens.

Pten^(i)pe−/−^ mice, generated by the ablation of the tumor suppressor gene Pten selectively in luminal prostatic epithelial cells at adulthood (*14*), faithfully recapitulate disease progression in humans. Within 3 months of Pten gene inactivation, prostates develop PINs, which then enter a latency phase where they exhibit characteristics of cellular senescence. At 12 months after Pten inactivation, some PINs evolve into adenocarcinoma (*15*). To comprehensively interrogate the effects of a vitamin D analog with reported anticancer activities in vivo (*16*) on prostatic precancerous lesions, we treated Pten^(i)pe−/−^ mice 9 months after Pten inactivation with Gemini-72. We show that this analog targets multiple microenvironmental aspects in Pten^(i)pe−/−^ prostates, including disrupting extracellular matrix (ECM) remodeling by stromal fibroblasts and modulation of myeloid-derived suppressor cell (MDSC) prostatic infiltration. Furthermore, it induces apoptosis in senescent PINs but eliminates only a minor subset of the most abundant epithelial cell population [luminal-C (Krt8/Krt4/Tacstd2)] in which interferon signaling is enhanced. Moreover, we show that antiapoptotic pathways are induced by Gemini-72 in persistent luminal-C cells. Therefore, our study unravels the diversity of vitamin D’s effects on various cell types of prostatic precancerous lesions and uncovers molecular mechanisms underlying the limitations of vitamin D–based chemoprevention and therapies.

## RESULTS

### Gemini-72 induces apoptosis of epithelial cells and normalizes ECM organization

**Fig. 1:**
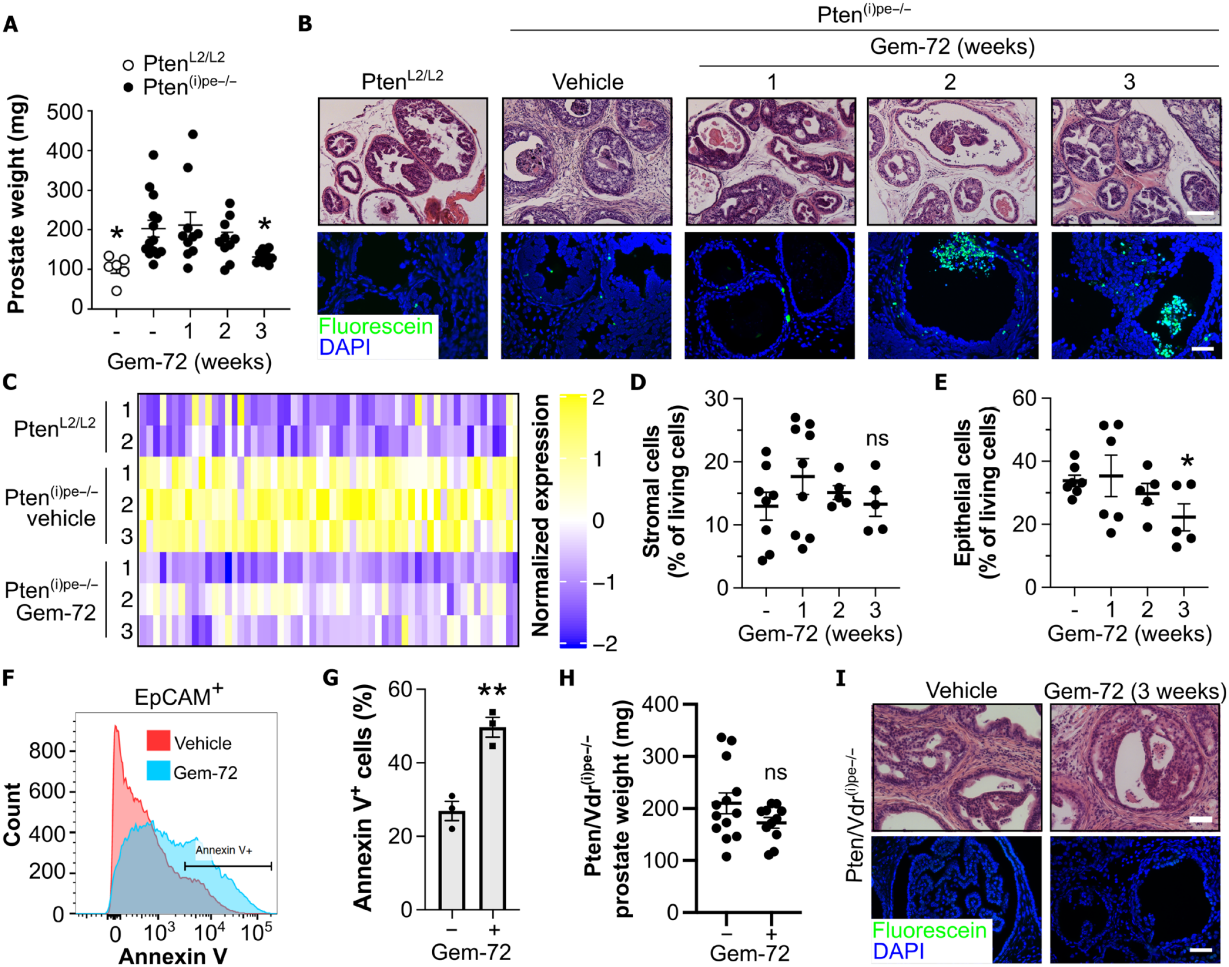
Effects of Gemini-72 on senescent PINs. (**A**) Prostate weight of vehicle-treated Pten^L2/L2^ mice (*n* = 6) and of Pten^(i)pe−/−^ mice treated with vehicle (*n* = 14) or Gemini-72 (Gem-72) for 1 to 3 weeks (*n* = 10 per condition). Means ± SEM. *P < 0.05 compared to vehicle-treated Pten^(i)pe−/−^ mice determined using one-way ANOVA followed by a post hoc Tukey test. (**B**) Hematoxylin and eosin (H&E) staining (top) and TUNEL assay (bottom) performed on prostatic sections of Pten^L2/L2^ and vehicle- and Gemini-72–treated Pten^(i)pe−/−^ mice. Representative images of the DL lobes are shown. *n* = 10 mice per condition. Scale bars, 100 μm. (**C**) Heat map of ECM organization–related genes in prostates of Pten^L2/L2^ mice and of Pten^(i)pe−/−^ mice treated for 3 weeks with vehicle or Gemini-72. Quantification of stromal (EpCAM^−^CD45^−^) (**D**) and epithelial (EpCAM^+^) cells (**E**) in prostates of vehicle- and Gemini-72–treated Pten^(i)pe−/−^ mice (*n* = 5 to 9) by flow cytometry. ns (not significant), *P* ≥ 0.05 compared to vehicle-treated mice. Representative FACS histogram plot (**F**) and quantification (**G**) of annexin V^+^ epithelial cells in Pten^(i)pe−/−^ mice treated for 1 week with vehicle or Gemini-72 (*n* = 3 per condition). Data were compared using Student’s *t* test. (**H**) Prostate weight of Pten/Vdr^(i)pe−/−^ mice treated with vehicle (*n* = 13) or Gemini-72 (*n* = 11) for 3 weeks. (**I**) Representative images of H&E-stained prostatic sections (top) and TUNEL assay (bottom). *n* = 10 mice per condition. Scale bars, 100 μm.

To investigate the impact of Gemini-72 on Pten-deficient PINs, we administered it to Pten^(i)pe−/−^ mice 9 months after gene inactivation (AGI). After 3 weeks of treatment (0.3 μg/kg per every other day), the prostate weight was reduced by 30% (Fig. 1A). Moreover, histological analyses revealed that the stromal reaction induced by Pten ablation was decreased after 1, 2, and 3 weeks of treatment (Fig. 1B, top). RNA sequencing (RNA-seq) of samples prepared from prostates of Pten^(i)pe−/−^ mice treated for 3 weeks with vehicle or Gemini-72 revealed that 2665 genes were differentially expressed (table S1). Reactome pathway analysis of these genes highlighted ECM organization as the major down-regulated pathway (table S2). Gemini-72 treatment reduced the transcript levels of >50 genes encoding proteins involved in ECM organization (e.g., collagen proteins and ECM remodeling enzymes) in prostates of Pten^(i)pe−/−^ mice to levels similar to those observed in wild-type mice (Fig. 1C and tables S1 and S2). Flow cytometry analysis revealed that Gemini-72 does not significantly affect the abundance of stromal cells (EpCAM^−^CD45^−^) in Pten^(i)pe−/−^ prostates after 3 weeks of treatment (Fig. 1D) but reduces the proportion of prostatic epithelial cells (EpCAM^+^) by 30% (Fig. 1E). Terminal deoxynucleotidyl transferase–mediated deoxyuridine triphosphate nick end labeling (TUNEL) assay on prostate sections from Pten^(i)pe−/−^ mice showed the presence of apoptotic cells in some PINs after 2- and 3-week Gemini-72 treatments (Fig. 1B, bottom). Moreover, fluorescence-activated cell sorting (FACS) analyses revealed that the proportion of annexin V^+^ epithelial cells (EpCAM^+^) was induced by twofold after 1 week of Gemini-72 treatment (Fig. 1, F and G). Because Pten-deficient PINs were senescence-associated β-galactosidase (SA-β-gal) positive, and multiplex cytokine analyses revealed the presence of high levels of various cytokines and chemokines in Pten^(i)pe−/−^ prostates, including many identified in the senescence-associated secretory phenotype [SASP; e.g., interleukin (IL)-1α, IL-1β, and CXCL1] (fig. S1, A to D), Gemini-72 may induce apoptosis of some senescent cells.

To determine whether the analog’s proapoptotic and anti-ECM effects were mediated by the vitamin D receptor (VDR) in luminal epithelial cells, Pten/Vdr^(i)pe−/−^ mice in which both Pten and Vdr are selectively inactivated in these cells at adulthood were treated at 9 months AGI with Gemini-72. A 3-week treatment of these mice did not reduce the prostate weight or stromal reaction (Fig. 1, H and I). Moreover, TUNEL assay showed that epithelial cells in PINs of Pten/Vdr^(i)pe−/−^ mice do not undergo apoptosis in response to treatment (Fig. 1I). Note that Gemini-72 did not induce apoptosis of senescent fibroblasts in vitro (fig. S2, A and B), indicating that the analog’s senolytic effect is cell type specific and/or context dependent. Together, these data show that prostatic epithelial VDR is required for the induction of apoptosis of some epithelial cells by the analog in senescent PINs and for the normalization of the stromal reaction.

### Gemini-72 affects numerous cell types including stromal fibroblasts

**Fig. 2:**
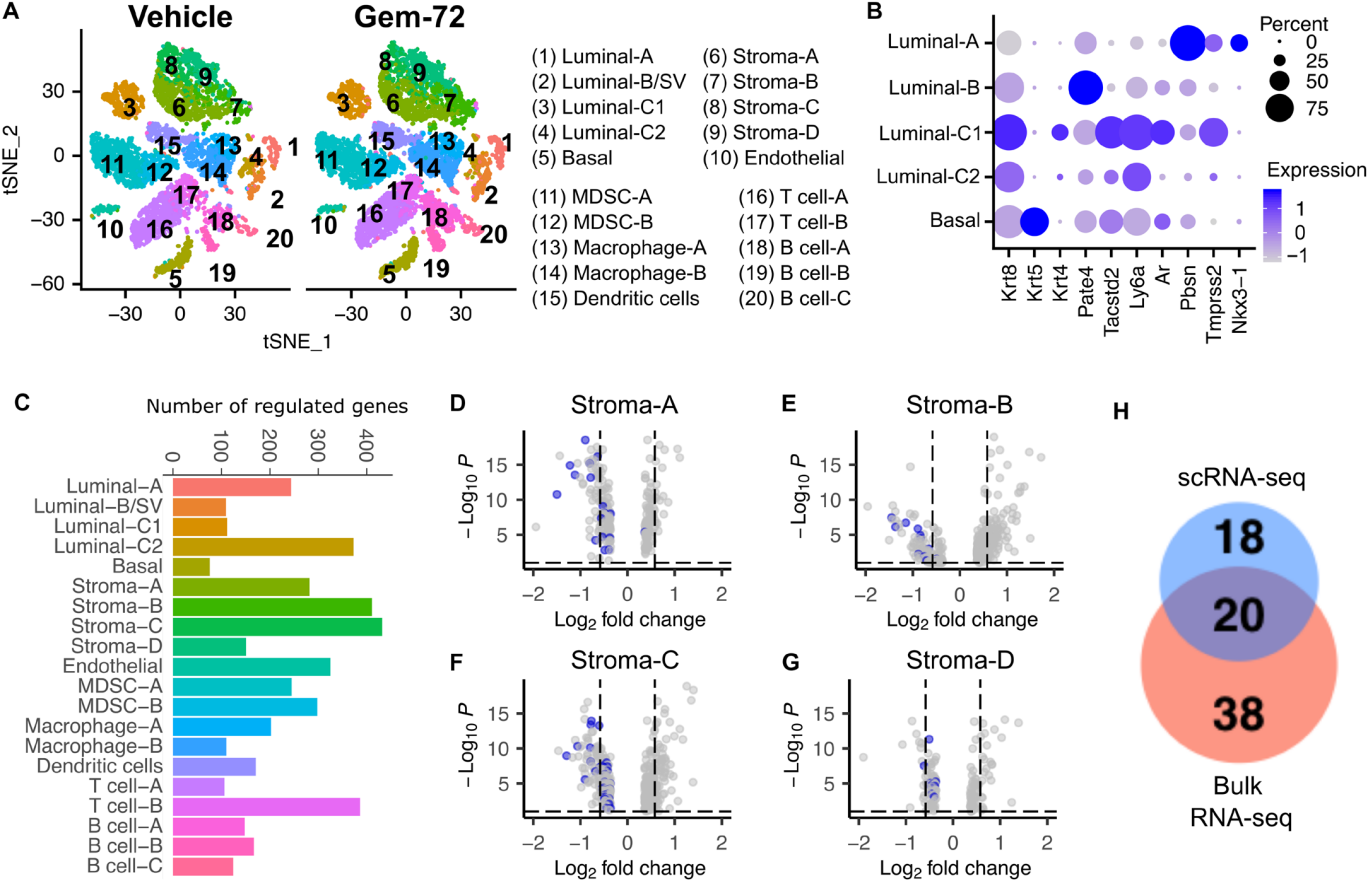
Impact of Gemini-72 on various cell populations in Pten^(i)pe−/−^ prostates. (**A**) t-SNE plot of cells from dissociated prostates of Pten^(i)pe−/−^ mice treated for 1 week with vehicle or Gemini-72 (left). Cell type annotations are indicated on the right. (**B**) Expression of epithelial cell lineage markers in the five epithelial clusters. (**C**) Bar chart depicting the number of deregulated genes by Gemini-72 in each cluster. (**D** to **G**) Volcano plots depicting genes modulated by Gemini-72 in the four stromal fibroblast clusters. Genes involved in ECM organization are highlighted in blue. (**H**) Venn diagram of Gemini-72–regulated, ECM organization–related genes identified by scRNA-seq of stromal fibroblasts (table S6) and by RNA-seq of whole prostates (table S2).

To investigate the effect of Gemini-72 on the various cell populations in the Pten^(i)pe−/−^ prostate in an unbiased and comprehensive manner, we performed droplet-based scRNA-seq on cells from dissociated prostates of Pten^(i)pe−/−^ mice treated at 9 months AGI for 1 week with vehicle or Gemini-72. On the basis of a t-distributed stochastic neighbor embedding (t-SNE) map generated with 5853 and 8662 cells from vehicle- and Gemini-72–treated mice, respectively, 20 cell clusters were identified in both conditions (Fig. 2A). Five were classified as epithelial (EpCAM) and 15 as mesenchymal (Vim). Among the latter, 10 were leukocytes (Ptprc) (fig. S3, A and B). The clusters were further characterized with additional cell lineage–specific markers (fig. S3C and table S3). The epithelial clusters comprised basal cells (Krt5; Cl-5) and two luminal clusters (Krt8; Cl-1 and Cl-2, denoted luminal-A and luminal-B, respectively) (Fig. 2B). Luminal-A cells express elevated levels of canonical androgen receptor (Ar) target genes Pbsn, Tmprss2, and Nkx3-1, whereas luminal-B cells express elevated levels of the epididymal gene Pate4 (Fig. 2B), indicating that these cells may originate from the seminal vesicle and have been included in the prostate sample preparation due to the anatomical closeness of the seminal vesicle to the prostate, as previously described (*11*). Two luminal cell clusters (Krt8) expressing Tacstd2 (Trop2), Ly6a (Sca-1), and Krt4 were identified (Cl-3 and Cl-4; denoted luminal-C1 and luminal-C2, respectively). Epithelial cells with similar profiles were shown to be present at low number in prostates of wild-type mice, but were the predominant epithelial population in Pten-deficient prostates (*17*). Furthermore, they have been identified by the FACS profile Lin^−^Sca-1^+^Cd49f^med^ [LSC^med^; (*17*)]. As clusters 3 and 4 express higher levels of Ly6a than luminal-A/B (Fig. 2B), and represent 60% of epithelial cells in Pten^(i)pe−/−^ prostates (table S4), the characteristics of luminal-C1 and luminal-C2 match those of LSC^med^ cells. Of note, the proportion of luminal-C1 cells expressing Ar and its target genes was higher compared to that of luminal-C2 cells (Fig. 2B).

The 10 identified leukocyte clusters (Ptprc) included B lymphocytes (Cd79a; Cl-18, C1-l9, and Cl-20), T lymphocytes (Cd3g; Cl-16 and Cl-17), macrophages (Adgre1; Cl-13 and Cl-14), MDSCs (S100a8; Cl-11 and Cl-12), and dendritic cells (Itgax; Cl-15) (fig. S3C). Nonleukocytic mesenchymal cells comprised endothelial cells (Pecam1; Cl-10) and stromal fibroblast clusters (Col1a1; Cl-6, Cl-7, Cl-8, and Cl-9) (fig. S3C).

In response to Gemini-72, 76 to 432 differentially expressed genes (DEGs) were identified in the various clusters, and stroma-B and stroma-C fibroblast clusters were those in which the larger numbers of genes were regulated (411 and 432, respectively) (Fig. 2C and table S5). Reactome pathway analysis of the DEG in the four stromal fibroblast clusters revealed that ECM organization was the top implicated pathway in the down-regulated genes of each cluster (20, 12, 31, and 9 genes in stroma-A, stroma-B, stroma-C, and stroma-D, respectively) (Fig. 2, D to G, and table S6), which is in agreement with our transcriptomic analysis on whole prostates and the attenuation of the stromal reaction by the analog (Fig. 1, B and C). Among the 38 ECM organization–related genes that were down-regulated by Gemini-72 in these stromal fibroblast clusters, 20 were shared with the down-regulated gene set identified in whole prostates of Gemini-72–treated mice, and included genes encoding collagen proteins [e.g., collagen, type I, α1 (Col1a1)], ECM proteins [e.g., fibronectin (Fn1) and fibulin 2 (Fbln2)], and ECM remodeling enzymes [e.g., matrix metalloproteinase 2 and 3 (Mmp2 and Mmp3)] (Fig. 2H and tables S1 and S6). Therefore, collectively, our results demonstrate the targeting of stromal ECM remodeling in Pten^(i)pe−/−^ prostates by Gemini-72.

### Gemini-72 differentially affects epithelial cells

**Fig. 3:**
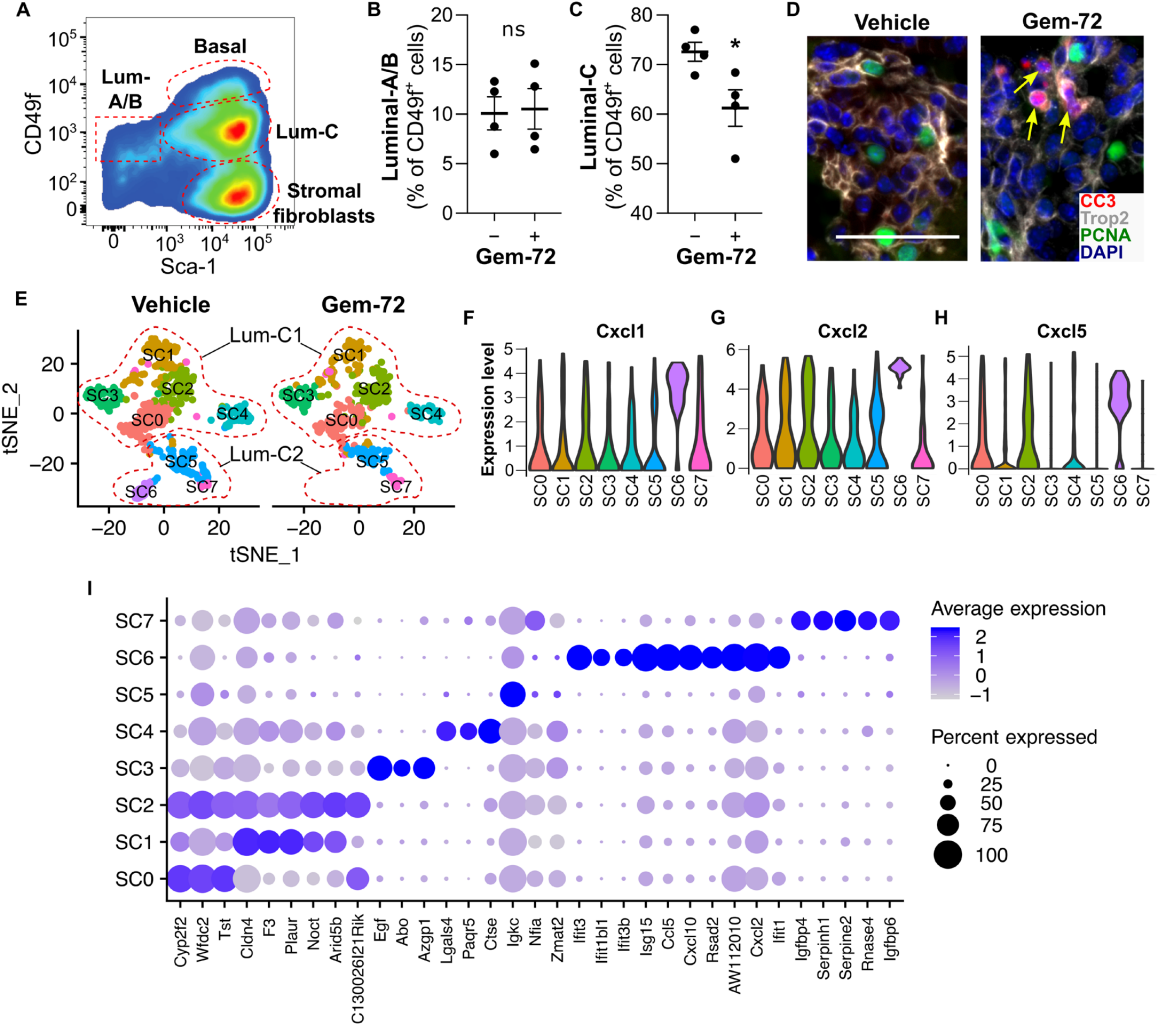
Effect of Gemini-72 on luminal-C subsets. (**A**) Representative pseudocolor plot of the cell populations identified by FACS in vehicle-treated Pten^(i)pe−/−^ mice (A) and quantification of luminal-A/B (**B**) and luminal-C (**C**) in Pten^(i)pe−/−^ mice treated for 1 week with vehicle and Gemini-72. Comparisons between vehicle- and Gemini-72–treated mice were performed using Student’s *t* test. Error bars ± SEM; *n* = 4 mice per condition. *P < 0.05; ns, *P* ≥ 0.05. (**D**) Representative Trop2 (gray), cleaved caspase 3 (CC3; red), PCNA (green) immunostainings, and DAPI (blue) in prostate sections of Pten^(i)pe−/−^ mice treated for 3 weeks with vehicle or Gemini-72. Representative images of the DL lobes are shown. *n* = 3 mice per condition. Scale bars, 50 μm. (**E**) t-SNE plot of luminal-C subclusters of a Pten^(i)pe−/−^ mouse treated for 1 week with vehicle or Gemini-72. Violin plots showing the expression of Cxcl1 (**F**), Cxcl2 (**G**), and Cxcl5 (**H**) in luminal-C subclusters of a vehicle-treated Pten^(i)pe−/−^ mouse. (**I**) Dot plot of subcluster-specific marker expression.

After Gemini-72 treatment, 76, 109, 112, 244, and 373 DEGs were identified in basal, luminal-B, luminal-C1, luminal-A, and luminal-C2 cells, respectively (Fig. 2C). Flow cytometry analyses revealed a 1.2-fold reduction in the proportion of luminal-C cells in Pten^(i)pe−/−^ mice treated with Gemini-72 for 1 week, whereas the proportion of luminal-A/B cells was similar in both conditions (Fig. 3, A to C). The number of Trop2-positive, cleaved caspase 3–positive PIN cells increased in prostate sections of Pten^(i)pe−/−^ mice after 3 weeks of Gemini-72 treatment (Fig. 3D). Because these cells were proliferating cell nuclear antigen (PCNA) negative, these results indicate that the analog induces apoptosis of luminal-C cells with characteristics of cellular senescence.

To further characterize the effects of Gemini-72 on luminal-C cells, we performed subcluster analysis. We identified eight subclusters (SC0 to SC7) in cells of the vehicle-treated Pten^(i)pe−/−^ mouse (Fig. 3E), five of which initially belonged to luminal-C1 (SC0 to SC4) and three to luminal-C2 (SC5 to SC7). These subclusters were also identified after Gemini-72 treatment, except SC6 (Fig. 3E). SC6 expressed much higher transcript levels of SASP components (i.e., Cxcl1, Cxcl2, and Cxcl5) than the others (Fig. 3, F to H) and of interferon-induced genes including *Ifit3*, *Rsad2*, and *Isg15* (Fig. 3I and table S7). Thus, these data indicate that these cells are highly responsive to Gemini 72 and are most likely eliminated via apoptotic cell death.

### NF-κB and Akt pathways are activated by Gemini-72 in persistent luminal-C cells

**Fig. 4:**
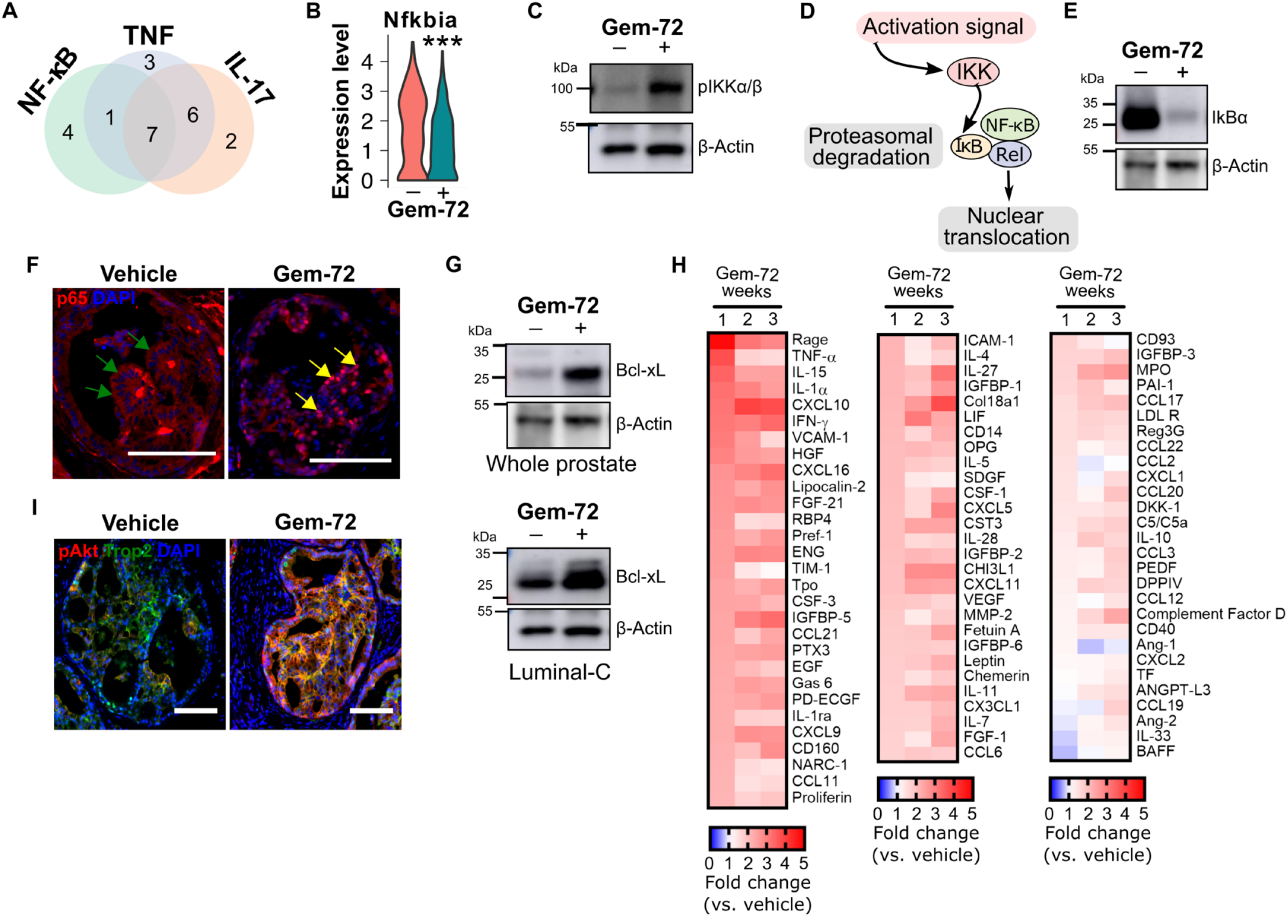
Prosurvival signaling in luminal-C cells of Pten^(i)pe−/−^ prostates in response to Gemini-72. (**A**) Venn diagram depicting the number of genes of the NF-κB, TNF, and IL-17 pathways downregulated in luminal-C cells of a Pten^(i)pe−/−^ mouse by a 1-week Gemini-72 treatment. (**B**) Violin plot showing the Nfkbia transcript levels in luminal-C cells of a Pten^(i)pe−/−^ mouse treated for 1 week with vehicle or Gemini-72. ***P < 0.001. Comparisons between vehicle- and Gemini-72–treated mice were performed using a Wilcoxon rank sum test. (**C**) Western blot analysis of phosphorylated IKKα/β^(S176/S180)^ (pIKKα/β) in pooled lysates of FACS-sorted luminal-C cells of three Pten^(i)pe−/−^ mice treated for 1 week with vehicle or Gemini-72. (**D**) Schematic representation of NF-κB activation. (**E**) Immunoblot of IκBα levels in whole prostates of Pten^(i)pe−/−^ mice treated with vehicle or Gemini-72 for 1 week. Lysates of three prostates per condition were pooled. (**F**) RelA/p65 (red) immunostaining in PINs of Pten^(i)pe−/−^ mice treated for 1 week with vehicle or Gemini-72. Green and yellow arrows point to cytosolic and nuclear p65 staining, respectively. Representative images of the DL lobes are shown. *n* = 3 mice per condition. Scale bars, 100 μm. (**G**) Western blot analysis of Bcl-xL in whole prostates (top) and FACS-sorted luminal-C cells (bottom) of Pten^(i)pe−/−^ mice treated for 1 week with vehicle or Gemini-72. *n* = 3 prostates per lane. (**H**) Heat map of cytokine levels in FACS-sorted luminal-C cells from Pten^(i)pe−/−^ mice treated at 9 months AGI with Gemini-72 for 1, 2, and 3 weeks. Lysates were obtained from a pool of three prostates per condition. (**I**) Phosphorylated Akt^(S473)^ (red) and Trop2 (green) immunostaining in PINs of Pten^(i)pe−/−^ mice treated for 3 weeks with vehicle or Gemini-72. Representative images of the DL lobes are shown. *n* = 3 mice per condition. Scale bars, 100 μm.

To gain insight into the effects of Gemini-72 on luminal-C cells, we determined the DEG in cells of the combined luminal-C1 and luminal-C2 clusters (table S8). KEGG (Kyoto Encyclopedia of Genes and Genomes) pathway analysis of genes down-regulated by Gemini-72 in these cells identified IL-17, tumor necrosis factor (TNF), and nuclear factor κB (NF-κB) signaling (Fig. 4A). A comparison of the gene lists revealed seven genes common among the three pathways, including Cxcl1 and Cxcl2, which are expressed at high levels by SC6 cells (Fig. 3, F and G). Because this subcluster is eliminated by Gemini-72, it is likely that the reduction in the transcript levels of these chemokines is due to the loss of this population. The negative regulator of NF-κB signaling, Nfkbia, which encodes the α isoform of the NF-κB inhibitor (IκB), was also among the common down-regulated genes (Fig. 4B and table S8). However, because the proportion of cells expressing Nfkbia in vehicle- and Gemini-72–treated luminal-C cells was similar (75 to 80%; table S8), the reduction in Nfkbia transcript levels was not due to the elimination of a particular cell subset. Furthermore, the levels of IKB kinase α/β phosphorylated at S176/180 (pIKKα/β^S176/180^) were markedly increased in response to Gemini-72 in FACS-sorted luminal-C cells (Fig. 4C). The phosphorylation of the IKK complex at these sites leads to the phosphorylation and subsequent degradation of IκB, enabling the nuclear translocation of the NF-κB heterodimer, where it regulates the expression of genes involved in inflammation (e.g., cytokines), cell proliferation, and survival (e.g., prosurvival Bcl2 family members) (Fig. 4D) (*18*). In agreement with the single-cell analysis, IκBα protein levels were strongly reduced by Gemini-72 in Pten^(i)pe−/−^ prostates (Fig. 4E). In addition, the cellular localization of RelA/p65, a member of the NF-κB transcription factor family, was mainly cytoplasmic in PIN cells of Pten^(i)pe−/−^ mice but nuclear in persistent cells after 1 week of Gemini-72 treatment (Fig. 4F). Moreover, the levels of the antiapoptotic factor Bcl-xL, which is an NF-κB target (*18*, *19*), were induced in response to Gemini-72 treatment in prostates of Pten^(i)pe−/−^ mice and in FACS-sorted luminal-C cells (Fig. 4G). Furthermore, a screen of 85 cytokines on FACS-sorted luminal-C cells revealed that the levels of 79 to 81 proteins were increased after 1, 2, and 3 weeks of treatment (Fig. 4H). As the expression of cytokines is controlled by NF-κB (*18*), these results indicate that NF-κB–cytokine/Bcl-xL antiapoptotic pathways are activated by Gemini-72 in luminal-C cells that persist after the treatment. In addition, the levels of Akt phosphorylated at S473, which are enhanced by Pten inactivation in prostatic luminal cells (*14*), were further increased in Trop2-positive PIN cells of Pten^(i)pe−/−^ prostates after 3 weeks of treatment (Fig. 4I). Collectively, our results show that protumoral signaling pathways, namely, Akt and NF-κB, are activated by Gemini-72 in persistent luminal-C cells.

### Gemini-72 transiently reduces MDSC prostatic infiltration in an epithelial Vdr-independent manner

**Fig. 5:**
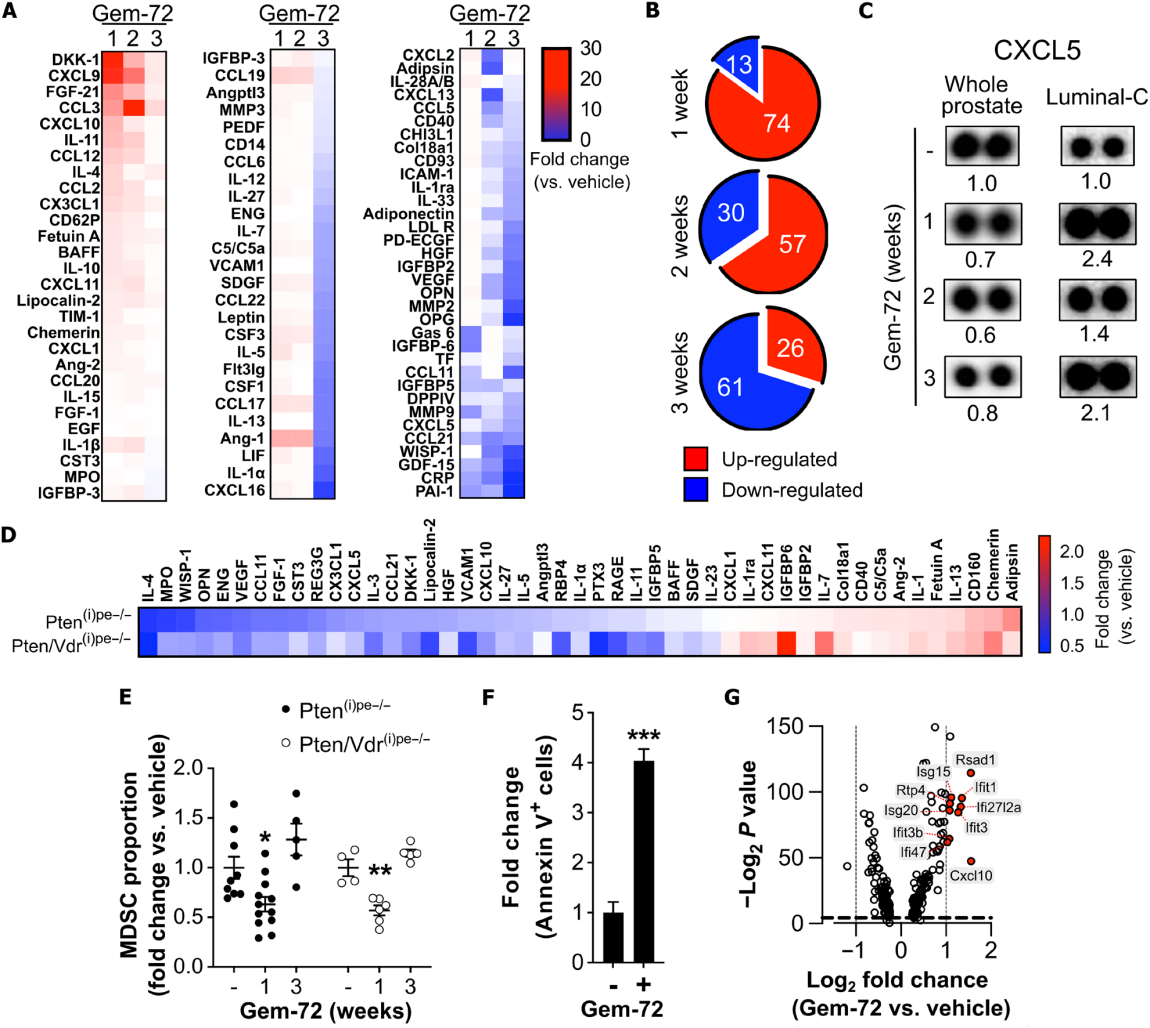
Effects of Gemini-72 on MDSCs in Pten^(i)pe−/−^ prostates. (**A**) Heat map of cytokine levels in whole prostates of Pten^(i)pe−/−^ mice treated with Gemini-72 for 1, 2, and 3 weeks. Lysates of three prostates per condition were pooled. (**B**) Pie charts depicting the number of up- and down-regulated cytokines in whole prostates after 1, 2, and 3 weeks of Gemini-72 treatment. (**C**) CXCL5 levels on cytokine array membranes of whole prostates and of luminal-C cells of Pten^(i)pe−/−^ mice after vehicle or 1, 2, and 3 weeks of Gemini-72 treatment. Lysates of three whole prostates per condition were pooled, and FACS-sorted luminal-C cells were obtained from a pool of three prostates. (**D**) Heat map of cytokine levels regulated by a 3-week Gemini-72 treatment in lysates of FACS-sorted stromal fibroblasts of Pten^(i)pe−/−^ and Pten/Vdr^(i)pe−/−^ prostates. Lysates were obtained from a pool of three prostates per condition. (**E**) FACS analysis of the proportion of MDSCs (CD11b^+^Gr-1^+^) in prostates of Pten^(i)pe−/−^ and Pten/Vdr^(i)pe−/−^ mice in response to Gemini-72 treatment. *n* = 4 to 12 mice per condition; error bars ± SEM. Comparisons between the different conditions were performed using one-way ANOVA followed by a post hoc Tukey test. *P < 0.05 and **P < 0.01, compared to vehicle-treated mice. (**F**) Quantification of annexin V^+^ leukocytes (CD45^+^) in prostates of Pten^(i)pe−/−^ mice treated for 1 week with vehicle or Gemini-72. *n* = 3 mice per condition. Data were compared using Student’s *t* test. ***P < 0.001. (**G**) Volcano plot depicting the genes significantly modulated by Gemini-72 in MDSC-A (cluster 11). Interferon signaling–related genes are highlighted in red.

Multiplex profiling of more than 80 cytokines in whole prostate lysates of Pten^(i)pe−/−^ mice revealed that the levels of 13, 30, and 61 of them were reduced after 1, 2, and 3 weeks of Gemini-72 treatment, respectively (Fig. 5, A and B). Pathway analysis (table S9) of the down-regulated proteins at 3 weeks of treatment demonstrated the targeting of ECM remodeling (e.g., MMP2, MMP9, and Col18a1) and endothelial factors (e.g., ICAM1, VCAM1, and VEGF), as well as IL-1 family members (IL-1α, IL-1β, IL-1ra, and IL-33). Moreover, the levels of CXCL5, a major MDSC chemoattractant (*20*), were reduced by Gemini-72 in whole prostates across all investigated time points (Fig. 5, A and C).

Because the scRNA-seq data demonstrated that the stromal fibroblasts of Pten^(i)pe−/−^ mice express a large number of cytokines and growth factors (table S3), we investigated whether Gemini-72 may have an impact on the stromal fibroblast secretome. Multiplex cytokine profiling illustrated a reduction in the levels of the MDSC recruiting factors CXCL5 and CX3CL1 in lysates of FACS-sorted stromal fibroblasts of prostates of Pten^(i)pe−/−^ and Pten/Vdr^(i)pe−/−^ mice treated for 3 weeks with Gemini-72 (Fig. 5D). Moreover, flow cytometric quantification of MDSCs in Pten^(i)pe−/−^ and Pten/Vdr^(i)pe−/−^ prostates demonstrated a reduction in their proportion in response to 1 week of Gemini-72 treatment (Fig. 5E), indicating that the analog’s impact on MDSC levels is epithelial cell VDR independent. Furthermore, we observed a fourfold increase in the number of annexin V^+^ leukocytes (CD45^+^) in Pten^(i)pe−/−^ prostates by a 1-week Gemini-72 treatment (Fig. 5F) and an induction of an interferon gene signature in MDSCs (cl-11) (Fig. 5G), which may be associated with apoptotic cell death. However, at 3 weeks of treatment, the proportion of MDSCs was similar to that of untreated mice (Fig. 5E). Thus, the down-regulation of CXCL5 by Gemini-72 might be counterbalanced at later time by the increase of other MDSC recruiting factors, e.g., CX3CL1 (Fig. 5A). Of note, CX3CL1 and CXCL5 levels were induced in luminal-C cells in response to 1, 2, and 3 weeks of Gemini-72 treatment (Figs. 4H and 5C), although luminal-C SC6 cells, which express high Cxcl5 levels (Fig. 3H), are eliminated. Together, these data indicate that the reduction in MDSC prostatic infiltration after 1 week of Gemini-72 treatment may be secondary to the regulation of stroma-derived factors that affect MDSC recruitment and/or due to a direct effect of the analog on this cell type.

## DISCUSSION

Although in vitro studies have attributed anticancer effects to vitamin D, clinical trials provided conflicting results (*5*, *7*). In this study, we comprehensively analyzed the effects of the vitamin D analog Gemini-72 on preneoplastic prostatic lesions of Pten^(i)pe/−^ mice. We show that in the convoluted microenvironment of Pten-deficient prostates, Gemini-72 elicits a complex, time-dependent, multifaceted cascade of activities and exerts disparate effects on distinct epithelial populations, as well as on the various cell types present in the microenvironment. Specifically, our detailed analyses identified various potent effects of Gemini-72, including attenuating the stromal reaction in prostates, by reducing the expression of ECM remodeling genes in stromal fibroblasts. These findings are in agreement with previous reports describing the targeting of cancer-associated fibroblasts (CAFs) by vitamin D analogs. Sherman *et al.* (*21*) demonstrated the regression of activated CAFs to a more quiescent state in a model of pancreatic cancer by the hypo-calcemic analog calcipotriol. Similarly, in colorectal cancer cells, calcitriol treatment was shown to inhibit the activation of CAFs, and VDR expression in fibroblasts of the tumor stroma correlated with increased overall survival in patients (*22*). However, our results show that epithelial VDR is essential for the inhibition of ECM remodeling in prostatic stromal fibroblasts by Gemini-72. Vitamin D signaling has also been reported to decrease the immunosuppressive capabilities of MDSCs through VDR in a cell-autonomous manner (*23*), and we demonstrate that Gemini-72 transiently reduces MDSC prostatic levels in an epithelial VDR-independent manner. Thus, Gemini-72 may have a direct effect on MDSCs. Together, our findings highlight the complex nature of cell-cell communication in prostatic tumors.

Our study also demonstrates multiple effects of Gemini-72 on epithelial subpopulations of senescent PINs. We show that treatment with the analog eliminates a minor population of luminal-C cells, characterized by high levels of the SASP chemokines Cxcl1 and Cxcl2, as well as interferon-regulated genes (SC6), via apoptotic cell death. This is of particular interest because Krt4/Tacstd2-expressing luminal cells have been shown to contribute to prostate regeneration after castration (*11*) and have superior organoid formation ability compared to other luminal cells under basal/precastration conditions (*11*, *12*). Our findings therefore indicate that Gemini-72 eliminates a subpopulation of luminal stem cells/progenitors implicated in the emergence of castration resistance.

In persistent luminal-C cells, Akt and NF-κB prosurvival signaling networks are activated by Gemini-72. The induction of NF-κB signaling in these cells may have consequences on both treatment outcome and disease progression. NF-κB activation culminates in the up-regulation of the antiapoptotic protein Bcl-xL, which may allow PIN cells to evade cell death by the analog. In addition, NF-κB–mediated cytokine induction in luminal-C cells may be responsible for the normalization of MDSC prostatic infiltration after initial inhibition by Gemini-72. As both MDSCs and activated NF-κB signaling are involved in the emergence of castration resistance (*24*, *25*), our results highlight potential mechanisms of treatment resistance in PIN cells that enable evasion of cellular demise by Gemini-72, which may explain the limited chemopreventive and therapeutic efficacy of vitamin D. Nonetheless, treatment of prostatic tumors with vitamin D analogs may sensitize resistant cells to other anticancer agents. We propose that Gemini-72 may enhance the sensitivity of luminal-C cells to a number of drugs targeting the prosurvival and antiapoptotic effects of NF-κB signaling, including the antidiabetic drug metformin and the Bcl2/Bcl-xL antagonist navitoclax (ABT-263). The former has been shown to inhibit SASP expression in a cellular model of oncogene-induced senescence through inhibiting NF-κB (*26*), whereas the latter induces apoptosis in senescent cells (*27*), which are known to resist apoptosis by up-regulating the NF-κB target gene Bcl-xL (*28*, *29*). Previous studies have highlighted the benefits of vitamin D and its analogs in combination chemotherapy (*30*). For instance, calcipotriol has been shown to enhance the antitumor efficacy of gemcitabine in the KPC mouse model of pancreatic cancer (*21*). Accordingly, a clinical trial (NCT03520790) investigating the potential benefits of combining the vitamin D analog paricalcitol with gemcitabine and nab-paclitaxel in the treatment of patients with metastatic pancreatic cancer has been initiated (*31*).

In conclusion, this comprehensive characterization outlines both the efficacy and limitations of vitamin D–based strategies in prostatic precancerous lesions. While the activity or abundance of different cell types in the microenvironment is affected by Gemini-72, prosurvival signaling networks are induced in persisting PIN cells. In light of these findings, we propose that while these therapies have modest efficacy in eliminating tumor cells, combinatorial strategies comprising vitamin D analogs may be beneficial in PCa prevention and treatment.

## MATERIALS AND METHODS

### Study design

The objective of our study was to comprehensively characterize the effects of the vitamin D analog Gemini-72 in prostatic precancerous lesions, identify the responses of the distinct cell types present, and shed light on mechanisms leading to tumor cell elimination and treatment resistance.

The experimental endpoint was a reduction in the prostate weight of Pten^(i)pe^ mice by Gemini-72. Sample size calculation was conducted for α = 0.05 and β = 0.8. As the mean (± SD) prostate weight of Pten^(i)pe−/−^ mice at 9 months AGI is 200 (± 65) mg, at least 10 mice are necessary to detect a 30% reduction in the prostate weight. We therefore treated 30 mice with Gemini-72 and sacrificed 10 after 1 week, 10 after 2 weeks, and 10 after 3 weeks. As the mean prostate weight of Pten/Vdr^(i)pe−/−^ mice is similar to that of Pten^(i)pe^ mice at 9 months AGI, 11 mice were treated with Gemini-72 for 3 weeks.

Animals included in this study were randomized to receive either vehicle or Gemini-72 (as described below). Investigators were not blinded to animal treatments or histological examination. However, the characterization of the analog’s effects on tumor weight, histology, and apoptotic induction (TUNEL assay) was performed by three investigators using three independent animal cohorts, each including three to five animals per condition. No outliers were excluded. Detailed descriptions of the different experimental methods used in this study, including the number of animals, are included in this study.

### Mice

Pten^(i)pe−/−^ mice have been generated as described (*14*, *15*). Briefly, mice harboring one copy of the PSA-CreER^T2^ transgene, expressing the tamoxifen-dependent CreER^T2^ recombinase under the control of the human PSA promoter in luminal cells of the prostatic epithelium, were intercrossed with mice in which Pten exons are flanked by LoxP sites (L2 allele), generating PSA-CreER^T2(tg/0)^/Pten^L2/L2^ (tg, transgenic) and PSA-CreER^T2(0/0)^/Pten^L2/L2^ mice. Pten/Vdr^(i)pe−/−^ mice were generated by intercrossing PSA-CreER^T2(tg/0)^/Pten^L2/L2^ mice with mice in which VDR alleles are floxed (*32*), generating PSA-CreER^T2(tg/0)^/Pten^L2/L2^/Vdr^L2/L2^ and PSA-CreER^T2(0/0)^/Pten^L2/L2^/Vdr^L2/L2^. Gene inactivation was induced in 8-week-old mice by intraperitoneal tamoxifen injections (1 mg per mouse) on five consecutive days. Genomic DNA was extracted from tail biopsies with a DirectPCR extraction kit (102-T; Viagen), and the various alleles were polymerase chain reaction (PCR)–amplified as described (*14*).

Breeding and maintenance of mice were carried out in the accredited Institut de génétique et de biologie moléculaire et cellulaire (IGBMC)/Institut Clinique de la Souris (ICS) animal house (C67-2018-37), in compliance with French and European Union regulations on the use of laboratory animals for research. All animal experiments were approved by the Ethical committee Com’Eth (Comité d’Ethique pour l’Expérimentation Animale, Strasbourg, France) and the French Ministry of Higher Education and Research (#3836-2016012818309429 v5).

### Gemini-72 treatment

Gemini-72 was a gift from H. Maehr. Mice were treated with 100 μl of Gemini-72, dissolved in sunflower oil at 0.3 μg/kg, or of sunflower oil (vehicle) every other day by oral gavage, for 1, 2, or 3 weeks.

### Histological examination

Whole prostates were fixed in 4% paraformaldehyde overnight at 4°C. Samples were embedded in paraffin, and 10-μm serial sections were cut. Sections were prepared for histological analysis by performing hematoxylin and eosin (H&E) staining, as per standard protocols.

### IMR-90 cells

IMR-90 cells were obtained from the American Type Culture Collection and cultured in Dulbecco’s modified Eagle’s medium (DMEM) [4.5 g/liter glucose, 10% fetal calf serum (FCS), and 1% penicillin/streptomycin (P/S)]. Senescence was induced in these cells by exposing them to X-ray irradiation at 10 Gy for 30 min. Cells were subsequently kept in a standard culture incubator (37°C, 5% CO_2_) with medium change every 2 days. At 10 days after irradiation, senescence state was evaluated by performing SA-β-gal staining. Irradiated cells were then treated with 100 nM Gemini-72 for 72 hours, and apoptotic induction was determined by cleaved caspase 3 immunoblotting. As positive controls of senolytic activity, cells were treated for 24 hours with ABT-263 (1 μM) or ABT-737 (1 μM).

### SA-β-gal staining

IMR-90 cells or frozen sections (10 μm) prepared from optimal cutting temperature (OCT) compound-embedded prostates were analyzed with the Senescence β-Galactosidase Staining Kit [Cell Signaling Technology (CST) #9860] following the manufacturer’s instructions. Sections were counterstained with hematoxylin and mounted before microscopic acquisition.

### TUNEL assay

Paraffin-embedded prostate sections (10 μm) were analyzed with the In Situ Cell Death Detection Kit, Fluorescein (reference: 11684795910; Roche) following the manufacturer’s instructions. Fluoromount-G Mounting Medium with 4′,6-diamidino-2-phenylindole (DAPI) (Invitrogen; 00-4959-52) was applied before microscopic acquisition.

### Immunostaining

Paraffin-embedded prostate sections (5 μm) were prepared and deparaffinized according to standard protocols. Heat-induced antigen retrieval was performed using a pressure cooker (20 min) and SignalStain Citrate Unmasking Solution (10×) (CST 14746). The following primary antibodies were used: phospho-Akt (S473) (CST 4060S; dilution 1:200), Trop2 (R&D Systems AF1122; dilution 1:50), cleaved caspase 3 (CST 9664; dilution 1:200), PCNA (Arigo Biolaboratories ARG62605; dilution 1:200), and RelA/p65 (CST 8242; dilution 1:400). The following secondary antibodies were used at a dilution of 1:400: Goat anti-Rabbit IgG (H+L) Highly Cross-Adsorbed Secondary Antibody, Alexa Fluor Plus 488 (Invitrogen; A32731), Goat anti-Mouse IgG (H+L) Highly Cross-Adsorbed Secondary Antibody, Alexa Fluor Plus 555 (Invitrogen; A32727), Donkey anti-Goat IgG (H+L) Highly Cross-Adsorbed Secondary Antibody, and Alexa Fluor Plus 647 (Invitrogen; A32849). Sections were mounted in Fluoromount-G Mounting Medium with DAPI (Invitrogen; 00-4959-52) before microscopic acquisition.

### Microscopic acquisition

Fluorescence and bright-field images were acquired using an upright motorized microscope (Leica DM 4000 B) fitted with the CoolSnap CF Color camera (Photometrics) and the Micro-Manager software, using the objectives 10× HC PL FLUOTAR (NA 0.30) and 20× HCX PL S-APO (NA 0.50). The Fiji software was used for image editing (*33*). Representative images were taken from the dorsolateral (DL) lobes, as the efficiency of the CreER^T2^-mediated gene inactivation is highest in these lobes (*14*).

### FACS analysis

#### Tissue dissociation

Prostate dissociation into single cells was performed following a previously described protocol (*34*), with minor modifications. Briefly, whole prostates were dissected, mechanically minced, and then enzymatically dissociated in DMEM [glucose (4.5 g/liter), GlutaMAX, 10% FCS, 1% P/S] containing collagenase type I (1 mg/ml) (17018029 Thermo Fisher Scientific/Gibco), with mild agitation for 45 to 60 min. Subsequently, trypsin/0.05% EDTA (Invitrogen/Gibco, catalog no. 25300) was added to the dissociated tissues for 5 min at 37°C. Cells were then resuspended in DMEM containing 500 U of recombinant ribonuclease (RNase)–free deoxyribonuclease (DNase) I (04716728001; Roche). Cellular clumps were dissociated into single cells by passing the suspension at least 10 times through a 20-gauge needle and once through a 40-μm cell strainer. The cell suspension was then washed once in phosphate-buffered saline (PBS).

#### Immunophenotyping experiments

Single cells isolated from a prostate were incubated with an anti-CD16/32 antibody (BD Pharmingen; 1:50) for 15 min on ice and with an antibody cocktail for 15 min on ice. The antibody cocktail included anti-CD326 (EpCAM) [BioLegend; reference: 118216; conjugation: phycoerythrin (PE)/Cy7], anti-CD45 (BioLegend; reference: 103128; conjugation: Alexa Fluor 700), anti-CD11b [eBioscience; reference: 45-0112-82; conjugation: peridinin chlorophyll protein (PerCP)–Cy5.5], and anti–Ly-6G/Ly-6C [eBioscience; reference: 11-5931-82; conjugation: fluorescein isothiocyanate (FITC)]. All antibodies were diluted 1:200 in DMEM [glucose (4.5 g/liter), 1% P/S, and 2% bovine serum albumin (BSA), without phenol red]. Samples were analyzed by a BD LSR II flow cytometer and the FlowJo software. The antibody cocktail enabled the quantification of epithelial cells (EpCAM^+^; CD45^−^), leukocytes (CD45^+^), stromal cells (EpCAM^−^CD45^−^), and MDSCs [CD45^+^CD11b^+^Ly-6G/Ly-6C (Gr-1)^+^].

#### FACS-sorting living cells for scRNA-seq

For scRNA-seq analysis, the prostate of one mouse per condition was dissociated and stained with DAPI. Living cells (DAPI^−^) were isolated with a BD FACSAria Fusion flow cytometer.

#### Annexin V assay

Individual prostates were dissociated and stained with anti-CD326 (EpCAM) (BioLegend; reference: 118216; conjugation: PE/Cy7) and anti-CD45 (BioLegend; reference: 103128; conjugation: Alexa Fluor 700) antibodies, diluted 1:200 in DMEM [glucose (4.5 g/liter), 1% P/S, and 2% BSA, without phenol red], and kept on ice for 15 min. Cells were washed once with PBS, resuspended in Annexin V Binding Buffer (BioLegend, reference: 422201) containing anti–annexin V antibody (BioLegend, reference: 640906; conjugation: FITC; dilution 1:20), and kept in the dark at room temperature for 15 min. Cells were washed once in PBS and resuspended in DMEM [glucose (4.5 g/liter), 1% P/S, 2% BSA, without phenol red] containing DAPI. Data were acquired using a BD LSR II flow cytometer and analyzed using the FlowJo software. Annexin V^+^ cells, independent of DAPI status, were quantified in epithelial cells (EpCAM^+^CD45^−^) and leukocytes (CD45^+^).

#### Prostatic epithelial subpopulation and stromal fibroblast sorting

Prostatic epithelial subpopulations were quantified as described (*17*). Briefly, individual prostates were dissociated and stained with the following antibodies purchased from eBioscience: CD31 (PECAM-1) (reference: 11-0311-85; conjugation: FITC; dilution 1:250), CD45 (reference: 11-0451-85; conjugation: FITC; dilution 1:250), TER-119 (reference: 11-5921-85; conjugation: FITC; dilution 1:250), CD49f (integrin α6) (reference: 12-0495-83; conjugation: PE; dilution 1:25), and Ly-6A/E (Sca-1) (reference: 17-5981-82; conjugation: APC; dilution 1:75), with DAPI. After selecting single, living cells, endothelial cells (CD31^+^), leukocytes (CD45^+^), and erythrocytes (TER-119^+^) were excluded, and epithelial subsets [luminal A/B (CD49f + Sca-1^−^); luminal-C (CD49f^med^Sca-1^−^); basal (CD49f^+^Sca-1^−^)] and stromal fibroblasts (CD49f^−^Sca-1^+^) were quantified. Data of individual mice were acquired using a BD LSR II flow cytometer and analyzed using the FlowJo software. To sort the epithelial subpopulations and stromal fibroblasts for subsequent protein analysis, dissociated prostates of three mice were pooled and sorted using a BD FACSAria Fusion flow cytometer.

### Western blotting

Whole prostates, FACS-sorted cells, and IMR-90 cells were lysed in ice-cold radioimmunoprecipitation assay buffer supplemented with protease (05892970001; Sigma-Aldrich) and phosphatase PhoSTOP (PHO SS-RO; Sigma-Aldrich) inhibitor cocktails. Protein concentrations of cell lysates were determined by Bradford assay (Abcam; ab119216). Equal amounts of proteins from samples were resolved by SDS–polyacrylamide gel electrophoresis and transferred onto nitrocellulose membranes (Trans-Blot Turbo Transfer System, Bio-Rad). Membranes were then incubated in blocking buffer (5% nonfat dry milk in tris-buffered saline/0.1% Tween 20) for 1 hour at room temperature and then probed with the following antibodies prepared at a dilution of 1:1000: Bcl-xL (CST 2764S), phospho-IKKα (Ser^180^)/IKKβ (Ser^181^) (CST 2681), IκBα (CST 4814), cleaved caspase 3 (CST 9664), β-actin (SCBT; sc-47778), histone H3 (CST; 4499S), and β-tubulin (IGBMC antibody facility, TUB-2A2). Anti-mouse immunoglobulin G (IgG) (CST 7076S) and anti-rabbit IgG (CST 7074S) horseradish peroxidase (HRP)–linked antibodies were used at a dilution of 1:5000. Lightning Plus-ECL, Enhanced Chemiluminescence Substrate (Perkin Elmer; reference: NEL104001EA) was used to visualize proteins on membranes, and images were captured using Amersham Imager 600 (GE Healthcare).

### Multiplex cytokine array

Levels of cytokines and growth factors were analyzed in lysates of FACS-sorted cells or of whole prostates using Proteome Profiler Mouse XL Cytokine Array (ARY028) and Proteome Profiler Mouse Cytokine Array Panel A (ARY006) kits, following the manufacturer’s instruction. Images of membranes were captured using Amersham Imager 600 (GE Healthcare) and analyzed using the Fiji software (*33*).

### Bulk RNA-seq and data analysis

Total RNA was isolated from a whole mouse prostate using the RNeasy Mini Kit (Qiagen). RNA quality was evaluated using Bioanalyzer 2100 (Agilent Technologies, Santa Clara CA), and samples with an RNA integrity number of >8 were analyzed. Sequencing was performed by the IGBMC GenomEast platform. Reads were mapped using TopHat v2.0.10 onto the mm10 assembly of mouse genome and the Bowtie2 v2.1.0 aligner (*35*). Reads uniquely aligned were used in further analyses. HTSeq-0.6.1 (*36*) was used for gene expression quantification. Normalization of read counts was performed across libraries, as previously proposed (*37*). Comparisons were performed using a previously described method (*38*), implemented in the DESeq2 Bioconductor library (DESeq2 v1.0.19). *P* values were adjusted for multiple testing. A gene was considered to be differentially expressed if the *P* value was less than 0.05 and the log_2_ fold change was >1. Pathway analysis of the genes was performed using the R package ReactomePA v 1.32.0 (*39*).

### scRNA-seq and data analysis

After prostate dissociation and cell sorting, cell number and viability were determined by a trypan blue exclusion assay on a Neubauer chamber. Samples contained >95% viable cells and were processed in parallel on the Chromium Controller from 10X Genomics (Leiden, The Netherlands). Ten thousand cells were loaded per well into nanoliter-scale Gel Beads-in-Emulsion (GEMs). Single-cell 3′ mRNA-seq library was generated according to Chromium Single Cell 3′ Reagent Kits User Guide (v2 Chemistry) from 10X Genomics (reference CG00052 Rev E). Briefly, GEMs were generated by combining barcoded gel beads, a reverse transcriptase master mix containing cells, and partitioning oil onto Chromium Chip A. Following full-length complementary DNA (cDNA) synthesis and barcoding from polyadenylated mRNA, GEMs were broken and pooled before cDNA amplification by 10 PCR cycles. After enzymatic fragmentation and size selection, sequencing libraries were constructed by adding P5 and P7 primers (Illumina, Paris, France) as well as sample index via end repair, A tailing, adaptor ligation, and PCR amplification with 12 cycles. Library quantification and quality control were performed using Bioanalyzer 2100 (Agilent Technologies, Santa Clara, CA). Libraries were then sequenced on Illumina HiSeq 4000 as 100-base paired-end reads. Image analysis, base calling, and demultiplexing were performed using RTA 2.7.7 and Cell Ranger 3.0.1 mkfastq. Alignment, barcode, and UMI filtering and counting were performed using Cell Ranger 3.0.1 count and mouse reference 3.0.0 (mm10 and Ensembl release 93). To obtain a matrix of the number of reads for each gene detected in each cell, the output of the Cell Ranger pipeline was read by the Read10X function of the Seurat v (version) 3.2 (*40*) R v 4.0.2. Genes expressed in less than 10 cells were excluded. Cells from a dissociated prostate of a vehicle- and Gemini-72–treated mouse with more than 100 and less than 5000 expressed genes and with less than 20% of mitochondrial genes were used to generate two Seurat objects. After log normalization (scale factor, 10,000), the two datasets were integrated (FindIntegrationAnchors, IntegrateData) by considering the principal component dimensions (PC) 1:30. This analysis resulted in an object of 14,515 cells collectively expressing 16,101 genes. Cluster analysis was performed using ScaleData, RunPCA (PC 1:10), and FindClusters with a 0.8 resolution. t-SNE (RunTSNE; PC 1:10, perplexity 30) was used as nonlinear dimensional reduction to visualize the data. Clusters containing more than 50 cells were further studied. Gene signatures were generated by the FindConservedMarkers function and then used to annotate the clusters. DEGs between vehicle- and Gemini-72–treated prostates were determined using the FindMarkers function in each cluster. Luminal-C subclustering was performed on a subset object composed of both luminal-C1 and luminal-C2 clusters using aforementioned functions, and subclusters with more than 20 cells were considered. Pathway analyses using Reactome and KEGG databases were performed with the R packages ReactomePA v 1.32.0 (*39*) and ClusterProfiler v 3.16.1 (*41*), respectively, and volcano plots were generated using the R package EnhancedVolcano v 1.6.0.

### Statistical analysis

Comparisons of data between two groups were done using Student’s *t* test and those between three and more by one-way analysis of variance (ANOVA) followed by post hoc analysis (Tukey’s test). Error bars depict SEM, and *P* values <0.05, 0.01, and 0.001 were indicated by *, **, and ***, respectively, whereas *P* values ≥0.05 were depicted as ns (not significant). The number of animals included per condition (*n*) is indicated in the figure legends.

## SUPPLEMENTARY MATERIALS

Supplementary material for this article is available at http://advances.sciencemag.org/cgi/content/full/7/31/eabg5982/DC1

## Appendix

View/request a protocol for this paper from *Bio-protocol*.
